# Supplementary material for: Short-term functional outcomes of patients with acute intracerebral hemorrhage in the native and expatriate population
Source: Front Neurol. 2024 May 1;15:1384985. doi: 10.3389/fneur.2024.1384985 (PMC11097684; doi:10.3389/fneur.2024.1384985)
Supplement: Supplementary file 1 [file Table_1.docx]

**Supplementary Table 1– comparison of features related with of ICH patients as defined by favorable (mRS 0-2) and unfavorable (mRS 3-6) outcome**

| **Variable** | **Favorable Prognosis**  **(n=939, 56.6)** | **Unfavorable Prognosis**  **(n= 721, 43.4)** | **Total (n=1660)** | **p value** |
| --- | --- | --- | --- | --- |
| Median (IQR) Age, years | 49.0 (36- 62) | 50.0 (31- 69) | 49.0 (41- 58) | 0.03 |
| Female, n (%) | 106 (14.7) | 175 (18.6) | 281 (16.9) | 0.03 |
| Male, n (%) | 615 (85.3) | 764 (81.4) | 1379 (83.1) |  |
| **Expatriate** |  |  |  |  |
| Native Residents n (%) | 141 (37.7) | 233 (62.3) | 374 (22.5) | 0.01 |
| South Asian n (%) | 402 (44.2) | 508 (55.8) | 910 (54.8) |  |
| Southeastern Asian n (%) | 144 (46.6) | 165 (53.4) | 309 (18.6) |  |
| African n (%) | 24 (44.4) | 30 (55.6) | 54 (3.3) |  |
| North American/European n (%) | 10 (76.9) | 3 (23.1) | 13 (0.8) |  |
| **Clinical Assessments** |  |  |  |  |
| Median Systolic blood pressure mmHg | 178 (127-229) | 184 (131-237) | 180 (153- 206) | 0.003 |
| Median Diastolic blood pressure mmHg | 106 (76- 136) | 108 (73-143) | 105.5 (90- 122) | 0.35 |
| Median Baseline NIHSS | 5 (1- 12) | 16 (1-31) | 11 (4- 20) | <0.001 |
| Median Glasgow Coma Scale | 15 (14-15) | 12 (8-14) | 14 (10- 15) | <0.001 |
| **GCS Severity** |  |  |  |  |
| GCS Score 13-15 | 647 (89.7) | 455 (48.5) | 1102 (66.4) | <0.001 |
| GCS Score 5-12 | 69 (9.6) | 349 (37.2) | 418 (25.2) |  |
| GCS Score 3-4 | 5 (0.7) | 135 (14.4) | 140 (8.4) |  |
|  |  |  |  |  |
| Median Baseline Blood Glucose, mg% | 7.0 (3.9- 10.1) | 8.0 (3.9- (12.1) | 7.5 (6.2- 9.9) | <0.001 |
| Median BMI* Kg/m^2^ | 26.7 (20.9- 32.5) | 27.1 (21.6- 32.6) | 26.9 (24.2-29.9) | 0.31 |
| **Concomitant Medications** |  |  |  |  |
| Antihypertensive use n (%) | 169 (23.4) | 225 (24.0) | 394 (23.7) | 0.8 |
| Anticoagulants use n (%) | 13 (1.8) | 36 (3.8) | 49 (3.0) | 0.02 |
| Antiplatelet use n (%) | 60 (8.3) | 130 (13.8) | 190 (11.4) | <0.001 |
| Antidiabetic use n (%) | 69 (9.6) | 121 (12.9) | 190 (11.4) | 0.04 |
| **Hemorrhage location** |  |  |  |  |
| Supratentorial Deep n (%) | 646 (68.8) | 437 (60.6) | 1083 (65.3) | <0.001 |
| Supratentorial Cortical n (%) | 117 (12.5) | 152 (21.1) | 269 (16.2) |  |
| Infratentorial n (%) | 164 (17.5) | 102 (14.1) | 266 (16.0) |  |
| Primary Intraventricular n (%) | 12 (1.2) | 30 ( (4.2) | 42 (2.5) |  |
|  |  |  |  |  |
| Any Intraventricular Hemorrhage n (%) | 166 (23.0) | 411 (43.8) | 577 (34.8) | <0.001 |
| Median Intraparenchymal Volume**, ml | 4.5 (2.3- 10.9) | 10.9 (4.2- 26.9) | 7.5 (3.2- 15.8) | <0.001 |
| Hemorrhage vol. ≥30 ml** | 22 (3.4) | 103 (11.9) | 125 (8.3) | <0.001 |
| **ICH score**** |  |  |  | <0.001 |
| 0 | 398 (62.3 | 251 (29.1) | 649 (43.2) |  |
| 1 | 203 (31.8) | 257 (29.8) | 460 (30.6) |  |
| 2 | 29 (4.5) | 195 (22.6) | 224 (14.9) |  |
| 3 | 7 (1.1) | 116 (13.5) | 123 (8.2) |  |
| 4 | 2 (0.3) | 39 (4.5) | 41 (2.7) |  |
| 5 | 0 | 4 (0.5) | 4 (0.3) |  |
|  |  |  |  |  |
| Stroke Unit care n (%) | 449 (62.3) | 277 (29.5) | 726 (43.7) | <0.001 |
| Intensive Unit Care n (%) | 226 (31.3) | 614 (65.4) | 840 (50.6) | <0.001 |
| Mechanical ventilation n (%) | 67 (9.3) | 497 (52.9) | 564 (34.0) | <0.001 |
| Extra-ventricular Drainage n (%) | 20 (2.8) | 98 (10.4) | 118 (7.1) | <0.001 |
| Craniotomy/Craniectomy n (%) | 14 (1.9) | 73 (7.8) | 87 (5.2) | <0.001 |
| **Disposition** |  |  |  |  |
| Discharged Home n (%) | 483 (67.0) | 68 (7.2) | 551 (33.2) | <0.001 |
| Admitted to Rehabilitation n (%) | 178 (24.7) | 397 (42.3) | 575 (34.6) |  |
| Transferred to long-term care n (%) | 4 (0.6) | 154 (16.4) | 158 (9.5) |  |
| Hospitalization for Other Comorbidities n (%) | 56 (7.8) | 161 (17.1) | 217 (13.1) |  |

* BMI values available in 1508 patients; ** Intraparenchymal Volume, Hemorrhage vol. ≥30 ml, ICH score is available in 1501

Supplementary Table 2- Characteristics and outcome patients with Intracerebral Hemorrhage on prior Anticoagulation

| **Characteristic or Investigation** | **Total**  **(n= 1660)** | **No Prior Anticoagulation (n= 1611, 97.0%)** | **With Prior Anticoagulation**  **(n= 49, 3.0%)** | **P-Value** |
| --- | --- | --- | --- | --- |
|  |  |  |  |  |
| **Median (IQR) Age, years** | **49 (41-58)** | **49 (41- 58)** | **66 (52- 77.5)** | **<0.001** |
| **Male Sex** | **1379 (93.1)** | **1345 (97.5)** | **34 (2.50)** | **0.01** |
| **Hypertension, n (%)** | **1383 (83.3)** | **1344 (97.2)** | **39 (2.8)** | **0.48** |
| **Diabetes, n (%)** | **565 (34.0)** | **533 (94.3)** | **32 (5.7)** | **<0.001** |
| **Dyslipidemia, n (%)** | **465 (28.0)** | **442 (95.1)** | **23 (4.9)** | **0.003** |
| **Prior Stroke, n (%)** | **113 (6.8)** | **104 (92.0)** | **9 (8.0)** | **0.001** |
| **Atrial Fibrillation, n (%)** | **55 (3.3)** | **27 (49.1)** | **28 (50.9)** | **<0.001** |
| **Coronary Artery Disease, n (%)** | **95 (5.7)** | **84 (88.4)** | **11 (11.6)** | **<0.001** |
| **Active Smoking, n (%)** | **172 (10.4)** | **169 (98.3)** | **3 (1.7)** | **0.32** |
|  |  |  |  |  |
| **Ethnicity** |  |  |  |  |
| **Native, n (%)** | **374 (22.5)** | **344 (92.0)** | **30 (8.0)** | **<0.001** |
| **South Asian, n (%)** | **910 (54.8)** | **897 (98.6)** | **13 (1.4)** |  |
| **Southeast Asian, n (%)** | **309 (18.6)** | **305 (98.7)** | **4 (1.3)** |  |
| **African, n (%)** | **54 (3.3)** | **52 (96.3)** | **2 (3.7)** |  |
| **Caucasian, n (%)** | **13 (0.8)** | **13 (100.0)** | **0** |  |
|  |  |  |  |  |
| **Median NIHSS on admission** | **11 (4- 20)** | **11 (4- 20)** | **11 (5.5- 21.5)** | **0.50** |
| **Median GCS on admission** | **14 (10- 15)** | **14 (10- 15)** | **14 (9.5- 15)** | **0.51** |
| **Median BMI on admission** | **27 (24.2- 30)** | **26.8 (24.2- 29.7)** | **29.4 (27- 33.4)** | **<0.001** |
| **Median RBS on admission** | **7.5 (6.2- 9.9)** | **7.5 (6.2- 9.9)** | **8.3 (6.3- 10.9)** | **0.15** |
| **HbA1c** | **6.5 ±1.7** | **6.5 ±1.7** | **6.9 ±1.8** | **0.18** |
| **Median Serum Cholesterol** | **4.5 (3.8- 5.3)** | **4.5 (3.9- 5.3)** | **3.9 (3.0- 4.9)** | **0.01** |
| **Median Serum Triglyceride** | **1.3 (1.0- 1.8)** | **1.3 (1.0- 1.8)** | **0.9 (0.8- 1.7)** | **0.06** |
| **Serum HDL** | **1.1 ±0.3** | **1.1 ±0.3** | **1.0 ±0.3** | **0.69** |
| **Serum LDL** | **2.9 ±15.9** | **2.9 ±1.3** | **2.4 ±1.0** | **0.07** |
| **Median Systolic Blood Pressure** | **180 (153—206)** | **180 (154- 207)** | **154 (137- 180)** | **<0.001** |
| **Diastolic Blood Pressure** | **106.5 ±24.9** | **107.1 ±24.9** | **89.1 ±19.2** | **<0.001** |
| **Median Heart Rate** | **82 (73- 94)** | **82 (73- 94)** | **82 (73.5- 98)** | **0.61** |
| **Median INR on Admission** | **1 (1.0 – 1.1)** | **1 (1.0- 1.1)** | **1.2 (1.0- 2.4)** | **<0.001** |
|  |  |  |  |  |
| **Site of Bleed** |  |  |  |  |
| **Subcortical** | **1072 (66.3)** | **1049 (97.9)** | **23 (2.1)** | **<0.001** |
| **Hemispheric** | **280 (17.3)** | **263 (93.9)** | **17 (6.1)** |  |
| **Brainstem** | **114 (7.0)** | **107 (93.9)** | **7 (6.1)** |  |
| **Cerebellum** | **152 (9.4)** | **151 (99.3)** | **1 (0.7)** |  |
|  |  |  |  |  |
| **Prognosis – At Discharge** |  |  |  |  |
| **Good (mRS 0-2)** | **511 (30.8)** | **503 (98.4)** | **8 (1.6)** | **0.03** |
| **Poor (mRS 3-6)** | **1149 (69.2)** | **1108 (96.4)** | **41 (3.6)** |  |
|  |  |  |  |  |
| **Prognosis – At 90-Days** |  |  |  |  |
| **Good (mRS 0-2)** | **738 (44.5)** | **725 (98.2)** | **13 (1.8)** | **0.01** |
| **Poor (mRS 3-6)** | **922 (55.5)** | **886 (96.1)** | **36 (3.9)** |  |
|  |  |  |  |  |
| **GCS Score on Admission** |  |  |  |  |
| **13-15** | **1102 (66.4)** | **1070 (97.1)** | **32 (2.9)** | **0.28** |
| **5-12** | **418 (25.2)** | **408 (97.6)** | **10 (2.4)** |  |
| **3-4** | **140 (8.4)** | **133 (95.0)** | **7 (5.0)** |  |
| **Characteristic or Investigation** | **Total**  **(n= 1660)** | **No Prior Anticoagulation (n= 1611, 97.0%)** | **With Prior Anticoagulation**  **(n= 49, 3.0%)** | **P-Value** |
|  |  |  |  |  |
| **30-Days Mortality** | **173 (10.4)** | **162 (93.6)** | **11 (6.4)** | **0.005** |
| **90-Days Mortality** | **251 (15.1)** | **237 (94.4)** | **14 (5.6)** | **0.008** |
|  |  |  |  |  |
| **Any Intraventricular Hemorrhage** | **577 (34.8)** | **559 (96.9)** | **18 (3.1)** | **0.77** |
| **ICH Volume ≥ 30 ml/cm^3^** | **125 (8.3)** | **120 (96.0)** | **5 (4.0)** | **0.43** |
| **Infratentorial Hemorrhage** | **227 (13.7)** | **219 (96.5)** | **8 (3.5)** | **0.58** |
| **Age ≥ 80 years** | **61 (3.7)** | **51 83.6)** | **10 (16.4)** | **<0.001** |
|  |  |  |  |  |
| **ICH Score** |  |  |  |  |
| **0** | **649 (43.2)** | **634 (97.7)** | **15 (2.3)** | **0.002** |
| **1** | **460 (30.6)** | **448 (97.4)** | **12 (2.6)** |  |
| **2** | **224 (14.9)** | **214 (95.5)** | **10 (4.5)** |  |
| **3** | **123 (8.2)** | **122 (99.2)** | **1 (0.8)** |  |
| **4** | **41 (2.7)** | **37 (90.2)** | **4 (9.8)** |  |
| **5** | **4 (0.3)** | **3 (75.0)** | **1 (25.0)** |  |
|  |  |  |  |  |
| **Median Hematoma Volume on First CT ml/cm^3^** | **7.5 (3.2—15.8)** | **7.5 (3.2- 15.8)** | **8.1 (2.7- 20.3)** | **0.94** |
| **Median Hematoma Volume on Second CT ml/cm^3^** | **8 (3.7- 17)** | **7.9 (3.8- 16.9)** | **8.9 (2.4- 19.5)** | **0.60** |
|  |  |  |  |  |

**IV- intravenous, NIHSS- National Institute of Health Stroke Scale, RBS – Random blood sugar, HDL- High density lipoprotein, LDL Low density lipoprotein, BMI- Body Mass Index, mRS- Modified Rankin Score, CABG- Coronary artery bypass Graft, PCI- Percutaneous Coronary Intervention, MACE- Major Cardiac Adverse Event**

Supplementary Table 3: Characteristics and outcome patients with and without antiplatelet medications

| **Characteristic or Investigation** | **Total**  **(n= 1660)** | **No Prior Antiplatelets**  **(n= 1470, 88.6%)** | **With Prior Antiplatelets**  **(n= 190, 11.4%)** | **P-Value** |
| --- | --- | --- | --- | --- |
| **Median Age, years** | **49 (41-58)** | **48 (41- 56)** | **61 (55- 74)** | **<0.001** |
| **Male Sex** | **1379 (83.1)** | **1223 (83.2)** | **156 (82.1)** | **0.71** |
| **Hypertension, n(%)** | **1383 (83.3)** | **1201 (81.7)** | **182 (95.8)** | **<0.001** |
| **Diabetes, n(%)** | **565 (34.0)** | **434 (29.5)** | **131 (68.9)** | **<0.001** |
| **Dyslipidemia, n(%)** | **465 (28.0)** | **357 (24.3)** | **108 (56.8)** | **<0.001** |
| **Prior Stroke, n(%)** | **113 (6.8)** | **74 (5.0)** | **39 (20.5)** | **<0.001** |
| **Atrial Fibrillation** | **55 (3.3)** | **36 (2.4)** | **19 (10.0)** | **<0.001** |
| **Coronary Artery Disease** | **95 (5.7)** | **38 (2.6)** | **57 (30.0)** | **<0.001** |
| **Active Smoking** | **172 (10.4)** | **151 (10.3)** | **21 (11.1)** | **0.74** |
|  |  |  |  |  |
| **Median NIHSS on admission** | **11 (4- 20)** | **11 (2—20)** | **11 (4- 20)** | **0.99** |
| **Median GCS on admission** | **14 (10- 15)** | **14 (10- 15)** | **14 (11- 15)** | **0.86** |
| **Median BMI on admission** | **27 (24.2- 30)** | **26.8 (24.2- 29.6)** | **27.3 (24.8- 32)** | **0.02** |
| **RBS on admission** | **8.8 ±3.9** | **8.6 ±3.8** | **10.3 ±4.6** | **<0.001** |
| **Median HbA1c** | **5.9 (5.4- 6.8)** | **5.8 (5.4- 6.6)** | **6.6 (5.7- 8.3)** | **<0.001** |
| **Serum Cholesterol** | **4.5 (3.8- 5.3)** | **4.6 (3.9- 5.4)** | **4.1 (3.2- 4.9)** | **<0.001** |
| **Serum LDL** | **2.9 ±1.2** | **2.9 ±1.3** | **2.3 ±0.9** | **<0.001** |
| **Systolic Blood Pressure** | **180.3 ±37.0** | **181.3 ±37.1** | **172.8 ±35.9** | **0.003** |
| **Median Hematoma Volume on First CT ml/cm^3^** | **7.5 (3.2—15.8)** | **7.5 (3.3- 15.7)** | **7.5 (2.7- 19.2)** | **0.88** |
| **Median Hematoma Volume on Second CT ml/cm^3^** | **8 (3.7- 17)** | **8 (3.7- 16)** | **7.4 (3- 21)** | **0.89** |
|  |  |  |  |  |
| **Site of ICH (n= 1618)** |  |  |  |  |
| **Subcortical** | **1072 (66.3)** | **965 (67.1)** | **107 (59.8)** | **0.02** |
| **Hemispheric** | **280 (17.3)** | **235 (16.3)** | **45 (25.1)** |  |
| **Brainstem** | **114 (7.0)** | **99 (6.9)** | **15 (8.4)** |  |
| **Cerebellum** | **152 (9.4)** | **140 (9.7)** | **12 (6.7)** |  |
|  |  |  |  |  |
| **GCS Score on Admission** |  |  |  |  |
| **13-15** | **1102 (66.4)** | **975 (88.5)** | **127 (11.5)** | **0.85** |
| **5-12** | **418 (25.2)** | **369 (88.3)** | **49 (11.7)** |  |
| **3-4** | **140 (8.4)** | **126 (90.0)** | **14 (10.0)** |  |
|  |  |  |  |  |
| **Any Intraventricular Hemorrhage** | **577 (34.8)** | **495 (33.7)** | **82 (43.2)** | **0.01** |
| **ICH Volume ≥ 30 ml/cm^3^** | **125 (8.3)** | **110 (8.2)** | **15 (9.1)** | **0.71** |
| **Infratentorial Hemorrhage** | **227 (13.7)** | **204 (13.9)** | **23 (12.1)** | **0.50** |
| **Age ≥ 80 years** | **61 (3.7)** | **41 (2.8)** | **20 (10.5)** | **<0.001** |
|  |  |  |  |  |
| **ICH Score (n=1501)** |  |  |  |  |
| **0** | **649 (43.2)** | **589 (44.1)** | **60 (36.4)** | **0.37** |
| **1** | **460 (30.6)** | **407 (30.5)** | **53 (32.1)** |  |
| **2** | **224 (14.9)** | **193 (14.4)** | **31 (18.8)** |  |
| **3** | **123 (8.2)** | **109 (8.2)** | **14 (8.5)** |  |
| **4** | **41 (2.7)** | **35 (2.6)** | **6 (3.6)** |  |
| **5** | **4 (0.3)** | **3 (0.2)** | **1 (0.6)** |  |
|  |  |  |  |  |
| **Prognosis – At 90-Days** |  |  |  |  |
| **Good (mRS 0-3)** | **987 (59.5)** | **902 (61.4)** | **85 (44.7)** | **<0.001** |
| **Poor (mRS 4-6)** | **673 (40.5)** | **568 (38.6)** | **105 (55.3)** |  |
|  |  |  |  |  |
|  |  |  |  |  |
| **Characteristic or Investigation** | **Total**  **(n= 1660)** | **No Prior Antiplatelets**  **(n= 1470, 88.6%)** | **With Prior Antiplatelets**  **(n= 11.4%)** | **P-Value** |
| **30-Days Mortality** | **173 (10.4)** | **145 (9.9)** | **28 (14.7)** | **0.04** |
| **90-Days Mortality** | **251 (15.1)** | **214 (14.6)** | **37 (19.5)** | **0.07** |
|  |  |  |  |  |
| **Size Of Hematoma (n= 1501)** |  |  |  |  |
| **< 10 ml/cm^3^** | **906 (60.4)** | **813 (60.9)** | **93 (56.4)** | **0.68** |
| **Between 10 – 19.9 ml/cm^3^** | **311 (20.7)** | **275 (20.6)** | **36 (21.8)** |  |
| **Between 20.0 – 29.9 ml/cm^3^** | **159 (10.6)** | **138 (10.3)** | **21 (12.7)** |  |
| **≥ 30.0 ml/cm^3^** | **125 (8.3)** | **110 (8.2)** | **15 (9.1)** |  |
|  |  |  |  |  |

Supplementary Table 4: The differences in the clinical risk factors and outcome between patients without and with diabetes

| **Characteristic or Investigation** | **Total**  **(n= 1660)** | **Non-Diabetics**  **(n=841, 50.7%)** | **Diabetics**  **(n= 565, 34%)** | **Pre-Diabetics**  **(n=254, 15.3%)** | **P-Value** |
| --- | --- | --- | --- | --- | --- |
| **Age, Mean, years** | **49 (41-58)** | **46 (39- 53)** | **56 (47- 65)** | **49 (43- 56)** | **<0.001** |
| **Male Sex** | **1379 (83.1)** | **709 (84.3)** | **456 (80.7)** | **214 (84.3)** | **0.18** |
| **Hypertension** | **1383 (83.3)** | **649 (77.2)** | **516 (91.3)** | **218 (85.8)** | **<0.001** |
| **Dyslipidemia** | **465 (28.0)** | **155 (18.4)** | **214 (37.9)** | **96 (37.8)** | **<0.001** |
| **Prior Stroke** | **113 (6.8)** | **35 (4.2)** | **69 (12.2)** | **9 (3.5)** | **<0.001** |
| **Atrial Fibrillation** | **55 (3.3)** | **15 (1.8)** | **34 (6.0)** | **6 (2.4)** | **<0.001** |
| **Coronary Artery Disease** | **95 (5.7)** | **22 (2.6)** | **65 (11.5)** | **8 (3.1)** | **<0.001** |
| **Active Smoking** | **172 (10.4)** | **94 (11.2)** | **55 (9.7)** | **23 (9.1)** | **0.52** |
|  |  |  |  |  |  |
| **Median NIHSS on admission** | **11 (4- 20)** | **12 (4.5- 21)** | **11 (4.5- 20)** | **8 (3- 16)** | **<0.001** |
| **Median GCS on admission** | **14 (10- 15)** | **14 (9- 15)** | **14 (10- 15)** | **15 (13- 15)** | **<0.001** |
| **Median BMI on admission** | **27 (24.2- 30)** | **26.3 (24- 29.4)** | **27.7 (24.5-31)** | **26.8 (24.7- 30)** | **<0.001** |
| **RBS on admission** | **8.8 ±3.9** | **7.6 ±2.6** | **11.2 ±5.0** | **7.3 ±1.9** | **<0.001** |
| **HbA1c** | **6.5 ±1.7** | **5.3 ±0.5** | **7.9 ±1.9** | **5.9 ±0.2** | **<0.001** |
| **Serum Cholesterol** | **4.6 ±1.2** | **4.6 ±1.0** | **4.5 ±1.4** | **4.8 ±1.0** | **0.04** |
| **Serum Triglyceride** | **1.6 ±1.2** | **1.4 ±0.9** | **1.8 ±1.6** | **1.6 ±0.9** | **<0.001** |
| **Serum HDL** | **1.1 ±0.3** | **1.1 ±0.3** | **1.0 ±0.3** | **1.1 ±0.4** | **0.006** |
| **Serum LDL** | **2.9 ±1.3** | **2.9 ±0.9** | **2.8 ±1.7** | **3.0 ±0.8** | **0.08** |
| **Systolic Blood Pressure** | **180.3 ±37.0** | **182.3 ±39.7** | **177.6 ±33.8** | **180.1 ±34.3** | **0.07** |
| **Diastolic Blood Pressure** | **106.5 ±24.9** | **109.2 ±25.9** | **101.9 ±24.5** | **108.0 ±21.1** | **<0.001** |
| **Median Hematoma Volume on 1st CT ml/cm^3^** | **7.5 (3.2—15.8)** | **8.4 (3.8- 17.3)** | **7 (2.9- 15.9)** | **5.6 (3.1- 11.2)** | **<0.001** |
| **Median Hematoma Volume on 2nd CT ml/cm^3^** | **8 (3.7- 17)** | **8 (4- 17.2)** | **7.2 (3.2- 18.5)** | **7.1 (3.5- 14.7)** | **0.12** |
|  |  |  |  |  |  |
| **Site of Bleed (n= 1618)** |  |  |  |  |  |
| **Subcortical** | **1072 (66.3)** | **530 (64.8)** | **357 (64.8)** | **185 (74.3)** | **0.04** |
| **Hemispheric** | **280 (17.3)** | **136 (16.6)** | **109 (19.8)** | **35 (14.1)** |  |
| **Brainstem** | **114 (7.0)** | **62 (7.6)** | **39 (7.1)** | **13 (5.2)** |  |
| **Cerebellum** | **152 (9.4)** | **90 (11.0)** | **46 (8.3)** | **16 (6.4)** |  |
|  |  |  |  |  |  |
| **GCS Score on Admission** |  |  |  |  |  |
| **13-15** | **1102 (66.4)** | **527 (47.8)** | **379 (34.4)** | **196 (17.8)** | **<0.001** |
| **5-12** | **418 (25.2)** | **223 (53.3)** | **150 (35.9)** | **(19.8** |  |
| **3-4** | **140 (8.4)** | **91 (65.0)** | **36 (25.7)** | **13 (9.3)** |  |
|  |  |  |  |  |  |
| **Intraventricular Hemorrhage** | **577 (34.8)** | **293 (34.8)** | **206 (36.5)** | **78 (30.7)** | **0.28** |
| **ICH Volume ≥ 30 ml/cm^3^** | **125 (8.3)** | **67 (8.9)** | **48 (9.4)** | **10 (4.3)** | **0.05** |
| **Infratentorial Hemorrhage** | **227 (13.7)** | **127 (15.1)** | **75 (13.3)** | **25 (9.8)** | **0.09** |
| **Age ≥ 80 years** | **61 (3.7)** | **21 (2.5)** | **34 (6.0)** | **6 (2.4)** | **0.001** |
|  |  |  |  |  |  |
| **ICH Score (n=1501)** |  |  |  |  |  |
| **0** | **649 (43.2)** | **308 (40.8)** | **218 (42.5)** | **123 (52.8)** | **0.02** |
| **1** | **460 (30.6)** | **237 (31.4)** | **149 (29.0)** | **74 (31.8)** |  |
| **2** | **224 (14.9)** | **117 (15.5)** | **83 (16.2)** | **24 (10.3)** |  |
| **3** | **123 (8.2)** | **66 (8.7)** | **47 (9.2)** | **10 (4.3)** |  |
| **4** | **41 (2.7)** | **25 (3.3)** | **14 (2.7)** | **2 (0.9)** |  |
| **5** | **4 (0.3)** | **2 (0.3)** | **2 (0.4)** | **0** |  |
|  |  |  |  |  |  |
| **30-Days Mortality** | **173 (10.4)** | **111 (13.2)** | **50 (8.8)** | **12 (4.7)** | **<0.001** |
| **90-Days Mortality** | **251 (15.1)** | **158 (18.8)** | **77 (13.6)** | **16 (6.3)** | **<0.001** |
|  |  |  |  |  |  |
| **Characteristic or Investigation** | **Total**  **(n= 1660)** | **Non-Diabetics**  **(n=841, 50.7%)** | **Diabetics**  **(n= 565, 34%)** | **Pre-Diabetics**  **(n=254, 15.3%)** | **P-Value** |
| **Prognosis – At 90-Days** |  |  |  |  |  |
| **Good (mRS 0-3)** | **987 (59.5)** | **481 (57.2)** | **324 (57.3)** | **182 (71.7)** | **<0.001** |
| **Poor (mRS 4-6)** | **673 (40.5)** | **360 (42.8)** | **241 (42.7)** | **72 (28.3)** |  |
|  |  |  |  |  |  |
| **Size Of Hematoma (n= 1501)** |  |  |  |  |  |
| **< 10 ml/cm^3^** | **906 (60.4)** | **426 (56.4)** | **311 (60.6)** | **169 (72.5)** | **<0.001** |
| **Between 10 – 19.9 ml/cm^3^** | **311 (20.7)** | **172 (22.8)** | **99 (19.3)** | **40 (17.2)** |  |
| **Between 20.0 – 29.9 ml/cm^3^** | **159 (10.6)** | **90 (11.9)** | **55 (10.7)** | **14 (6.0)** |  |
| **≥ 30.0 ml/cm^3^** | **125 (8.3)** | **67 (8.9)** | **48 (9.4)** | **10 (4.3)** |  |
|  |  |  |  |  |  |
| **Ethnicity** |  |  |  |  |  |
| **Native** | **374 (22.5)** | **134 (35.8)** | **198 (52.9)** | **42 (11.2)** | **<0.001** |
| **South Asian** | **910 (54.8)** | **475 (52.2)** | **286 (31.4)** | **149 (16.4)** |  |
| **Southeast Asian** | **309 (18.6)** | **193 (62.5)** | **61 (19.7)** | **55 (17.8)** | **<0.001** |
| **African** | **54 (3.3)** | **32 (59.3)** | **16 (29.6)** | **6 (11.1)** |  |
| **Caucasian** | **13 (0.8)** | **7 (53.8)** | **4 (30.8)** | **2 (15.4)** |  |
|  |  |  |  |  |  |
